# Effects of Light Quality on Flowering and Physiological Parameters of *Cymbidium ensifolium* ‘Longyan Su’

**DOI:** 10.3390/plants14233670

**Published:** 2025-12-02

**Authors:** Luyu Xue, Yanru Duan, Xiuling Li, Chenye Li, Xiuming Chen, Fei Wang, Yulu Ji, Jinliao Chen, Yu Jiang, Zifu Liu, Ning Liu, Donghui Peng

**Affiliations:** 1 The Cross-Strait Scientific and Technological Innovation Hub of Flower Industry, Key Laboratory of National Forestry and Grassland Administration for Orchid Conservation and Utilization at College of Landscape Architecture and Art, The Innovation and Application Engineering Technology Research Center of Ornamental Plant Germplasm Resources in Fujian Province, National Long Term Scientific Research Base for Fujian Orchid Conservation, Fujian Agriculture and Forestry University, Fuzhou 350002, China; luyuxue@fafu.edu.cn (L.X.); yanruduan@fafu.edu.cn (Y.D.); congzxiao@163.com (X.L.); chenyeli@fafu.edu.cn (C.L.); cxm291802145@fafu.edu.cn (X.C.); wangfei_icon@163.com (F.W.); july55775@163.com (Y.J.); fjchenjl@fafu.edu.cn (J.C.); 2 Institute of Horticulture, Sichuan Academy of Agricultural Sciences, Chengdu 610000, China; iamjiangyu@163.com; 3 Wuping County Forestry Bureau, Longyan 364300, China; liuwj2729@163.com

**Keywords:** *Cymbidium ensifolium* ‘Longyan Su’, light quality, flowering time, flower quality, physiological mechanism

## Abstract

As a highly valued orchid species, *Cymbidium ensifolium* (*C. ensifolium*) exhibits a natural flowering period mainly from July to September, which does not align with the market demand and shows low flowering quality, thereby significantly constraining the development of the *C. ensifolium* floriculture industry. To address this key issue, the study used *C. ensifolium* ‘Longyan Su’ as the experimental material, with white light as the control and composite light with varying ratios of red and blue light as the treatments, and investigated the influence of light quality on flowering. The results showed that blue light could significantly advance the flowering time, while red light could markedly improve the flower quality. Blue light promoted the accumulation of soluble protein and soluble sugar during flower bud differentiation, while red light enhanced their accumulation during floral organ development. During the flower bud differentiation and development stage, blue light increased the synthesis of abscisic acid (ABA) in leaves, and red light promoted the production of gibberellic acid (GA_3_) and zeatin riboside (ZR). The study provides an important foundation and reference for further analysis of the flowering mechanism of *C. ensifolium* under different light quality treatments, and also provides technical support for flowering regulation of orchids in practical production.

## 1. Introduction

*Cymbidium ensifolium*, one of the traditional orchids of China, also known as the “Four Seasons Orchid”, belongs to the Orchidaceae family and the *Cymbidium* genus. Its rich fragrance, elegant foliage, high ornamental value, and long history of cultivation contribute to its popularity among consumers [1,2]. ‘Longyan Su’ is a commonly cultivated variety of *C. ensifolium*, named after its origin in Longyan, Fujian, China, and is one of the most widely circulated and renowned pure-lip cultivars. ‘Longyan Su’ is cherished by scholars and connoisseurs for its graceful foliage and delicate floral hues. However, its natural flowering period, which mainly occurs from July to September, is misaligned with major Chinese traditional festivals such as National Day and the Spring Festival, and its suboptimal flower quality severely hinders the industrial development of pure-lip *C. ensifolium*. Therefore, mastering the artificial regulation of the flowering period in *C. ensifolium* ‘Longyan Su’ not only aligns its blooming season with market demand but also improves flower quality, which is crucial for the development of the *C. ensifolium* industry.

Light functions as a regulatory signal in plant morphogenesis, photosynthesis, and seed dormancy [3,4]. Among these, light quality is essential for plant growth and development, as it can alter the plant’s morphological appearance, biochemical characteristics, photosynthetic efficiency, and fruit quality [5]. Research has shown that compared with other light qualities such as green light and yellow light, red light and blue light are more beneficial for plant growth [6]. Therefore, many recent studies have focused on red and blue monochromatic lights, as well as their combinations in varying ratios. The responses of plants to different light qualities are mediated by the corresponding photoreceptors. These photoreceptors are broadly classified into phytochromes, cryptochromes, phototropins, and UV-B receptors. Among them, phytochromes are the primary receptors for red and far-red light, whereas cryptochromes mainly perceive blue and near-ultraviolet light. Multiple phytochromes, including phytochrome A (PHYA), phytochrome B (PHYB), phytochrome C (PHYC), phytochrome D (PHYD), and phytochrome E (PHYE), as well as cryptochrome 1 (CRY1) and cryptochrome 2 (CRY2), have been identified in different plant species [7], and together they regulate the flowering process by modulating gene expression, maintaining hormone homeostasis, and influencing other key physiological pathways. Photoreceptor-mediated hormonal regulation plays a key role in the regulation of flowering in response to light quality. Blue light can suppress the biosynthesis and signaling of indole-3-acetic acid (IAA) and gibberellic acid (GA_3_) through the cryptochrome signaling pathway, while simultaneously activating transcriptional pathways related to abscisic acid (ABA) biosynthesis and transport, thereby promoting ABA accumulation [8]. In contrast, red light increases gibberellin (GA) levels and decreases ABA levels via the phytochrome PHYB pathway [9]. It should be noted that these hormonal changes exert different influences on vegetative and reproductive growth across various plant materials. In addition, red light can induce phytochrome interconversion and enhance carbon–nitrogen metabolism, vegetative growth, and flowering [10], whereas blue light can regulate protein synthesis, chloroplast development, and the flowering process [11].

Both red and blue monochromatic lights as well as their composite lights with different ratios exert a positive effect on plant flowering, including advancing flowering time, increasing flower bud germination rates, improving flower quality, and extending flowering periods. Numerous studies have reported the regulatory effects of red and blue light on plant flowering. For example, under an R:B ratio of 9:1, *Lachenalia* ‘Rupert’ exhibited noticeably elongated inflorescences and an increased number of florets [12]; red light exposure significantly promoted the formation and development of flowers in *Ipomoea nil* and *Antirrhinum majus* [13]; red-blue composite light was beneficial for the flowering and flower development of *Cyclamen persicum* [14]. Moreover, exposure to blue light enhanced flowering and resulted in a higher number of flowers in *Crocus sativus* [15]. Within the Orchidaceae family, most existing research has concentrated on genera of higher economic value. After treatment with different monochromatic LED lights, it was found that red light and a high proportion of red light could advance the flowering time of *Phalaenopsis*, increase the number of florets, and enhance the inflorescence height [16]. For *Dendrobium officinale* tissue-cultured seedlings, an R:B:G ratio of 4:2:1 promoted earlier flowering and increased flowering rates [17]; *Dendrobium nobile* ‘Zixia’ showed the highest nutrient content and greatest environmental stress tolerance under an R:B ratio of 8:2 [18]; *Dendrobium denneanum* extended its flowering duration under an R:B ratio of 1:3 [19]. These studies have predominantly focused on *Phalaenopsis* and *Dendrobium*, while research on *Cymbidium* is relatively limited. Therefore, it is particularly important to systematically investigate the regulatory mechanisms by which light quality influences flowering in *C. ensifolium*.

Our group’s previous research demonstrated that blue light significantly advanced flowering time, increased flower bud induction rates, and enhanced the proportion of normal flowers in *C. ensifolium* tissue-cultured seedlings. However, tissue-cultured seedlings exhibit differences from seed-derived plants in developmental stages and physiological foundations. To validate the applicability of this light quality regulation pattern to seedling-grown plants, this study used white light as the control, along with red and blue monochromatic lights, and composite lights with varying ratios of these two colors as treatment groups, to explore their effects on flower bud differentiation and flowering quality in three-year-old *C. ensifolium* ‘Longyan Su’ plants. The aim is to identify the optimal red-blue light ratio for flowering in *C. ensifolium*, master the techniques for artificial regulation of flowering in ‘Longyan Su’, enhance its ornamental and economic value, provide theoretical foundations and practical strategies for the flowering regulation of orchids, and offer technical support for the rapid development of the orchids industry.

## 2. Results

### 2.1. Effects of Light Quality on Flowering Time and Inflorescence Flowering Duration in C. ensifolium

**Figure 1:**
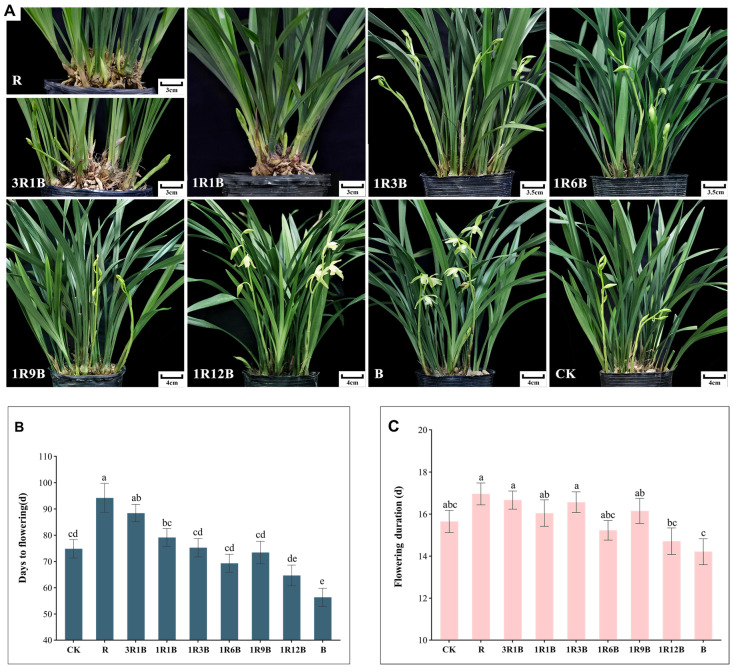
Effects of different treatments on flowering time and inflorescence flowering duration in *C. ensifolium* ‘Longyan Su’. (**A**) Image showing the flowering of ‘Longyan Su’ in different treatments after 68 days of treatment, (**B**) the number of days required for flowering, (**C**) the flowering duration of inflorescence. Values in the figure represent the mean ± SE (*n* = 15). Distinct letters indicate treatments that differ significantly (*p* < 0.05, Duncan’s test).

The effects of different treatments on the flowering time and flowering duration of *C. ensifolium* ‘Longyan Su’ were analyzed (Figure 1). The results indicated that under the B treatment, flowering time was significantly advanced, with 56 days required from the beginning of treatment to flowering, which was 18 days earlier than the control (Figure 1A,B). In contrast, both the R and 3R1B treatments significantly delayed flowering, with the R treatment showed the latest flowering time, which required 94 days from the beginning of treatment to flowering and was 19 days longer than the control (Figure 1A,B); in addition, flowering was delayed by 13 days under the 3R1B treatment (Figure 1A,B). Flowering duration of inflorescences in each treatment did not differ significantly from the control (Figure 1C). In summary, it was found that under the red-blue composite light, with the proportion of blue light gradually increasing, the flowering time of ‘Longyan Su’ was continuously advanced; in contrast, with the proportion of red light gradually increasing, the flowering time was progressively delayed. Specifically, blue light significantly advanced the flowering time of ‘Longyan Su’, whereas red light delayed flowering time of ‘Longyan Su’. In addition, red light slightly prolonged the flowering period, and the extension effect was more pronounced when the proportion of blue light was lower and that of red light was higher; however, overall, the effect was not statistically significant.

### 2.2. Effect of Light Quality on the Flower Quality of C. ensifolium

**Figure 2:**
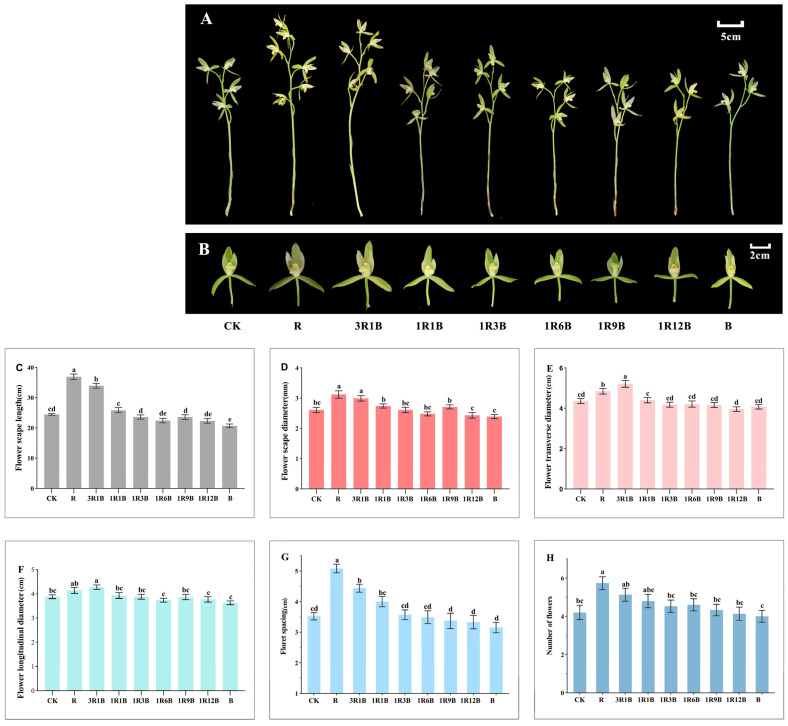
Effects of different treatments on the flowering traits of *C. ensifolium* ‘Longyan Su’. (**A**) Image showing the flower scapes of ‘Longyan Su’ under different treatments, (**B**) Image showing the flower size of ‘Longyan Su’ under different treatments, (**C**) flower scape length, (**D**) flower scape diameter, (**E**) flower transverse diameter, (**F**) flower longitudinal diameter, (**G**) floret spacing, (**H**) number of flowers per flower scape. Values in the figure represent the mean ± SE (*n* = 15). Distinct letters indicate treatments that differ significantly (*p* < 0.05, Duncan’s test).

This study conducted statistical analyses on relevant flower quality traits, including flower scape length, flower scape diameter, flower transverse diameter, flower longitudinal diameter, floret spacing, and number of flowers per flower scape for each treatment group of *C. ensifolium* ‘Longyan Su’. The results indicated that light quality treatments had significant effects on all of these indicators (Figure 2). The R and 3R1B treatments markedly improved flower quality in ‘Longyan Su’. The R treatment was the most effective for the entire flower scape, with a flower scape length of 36.90 cm, a flower scape diameter of 3.12 mm, a flower transverse diameter of 4.84 cm, a flower longitudinal diameter of 4.14 cm, and the total number of flowers per flower scape was 5.73 (Figure 2A). For individual flowers, the 3R1B treatment showed the best effect, with a flower transverse diameter of 5.21 cm and a flower longitudinal diameter of 4.28 cm, which indicated that flower quality was the highest under the 3R1B treatment (Figure 2B,E,F). Under the B treatment, with a flower scape length of 20.67 cm, a flower scape diameter of 2.39 mm, a flower transverse diameter of 4.07 cm, a flower longitudinal diameter of 3.63 cm, respectively, and the total number of flowers per flower scape was 4, no significant differences in flower quality were observed compared with the control (Figure 2A,B). In summary, the blue light treatment showed no significant difference in flower quality compared with the control, while the red light treatment significantly improved flower quality and enhanced the ornamental value of *C. ensifolium* ‘Longyan Su’.

The integration of the results from Section 2.1 and Section 2.2 showed that blue light treatment could significantly advance the flowering time of *C. ensifolium* ‘Longyan Su’, but it had no significant effect on flower quality; while red light treatment could delay the flowering time of *C. ensifolium* ‘Longyan Su’, effectively improved flower quality and enhanced its ornamental value.

### 2.3. Effects of Light Quality on on Chlorophyll Content in C. ensifolium Leaves

Based on the results of the above analyses, the control group (CK) and five treatments with significant phenotypic differences—R, 3R1B, 1R1B, 1R12B, B—were selected for further determination and analysis of physiological parameters, including chlorophyll, soluble protein, soluble sugar, and endogenous hormones.

**Figure 3:**
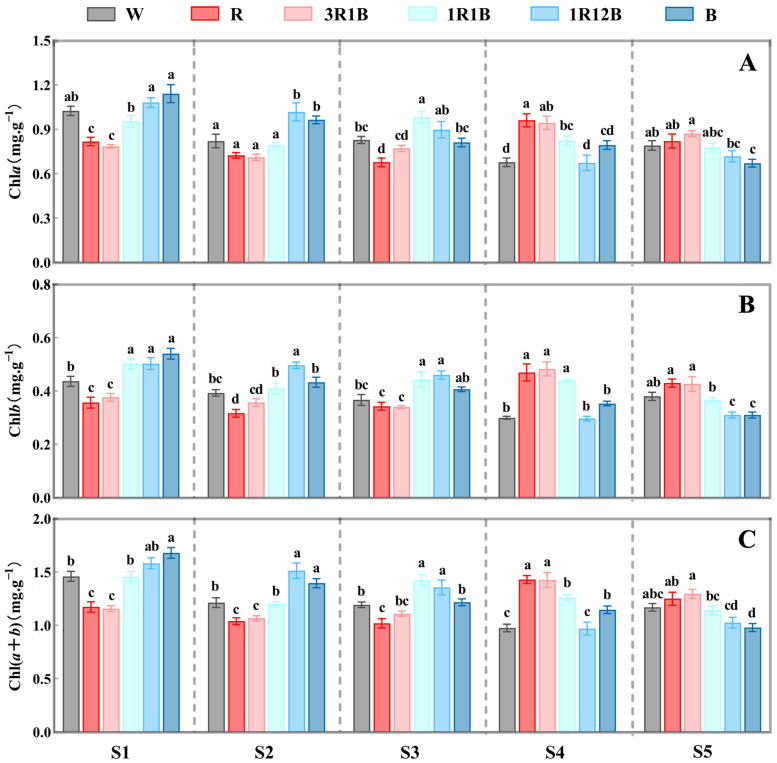
Effects of different treatments on chlorophyll content in the leaves of *C. ensifolium* ‘Longyan Su’. (**A**) Chlorophyll *a*, (**B**) Chlorophyll *b*, (**C**) Chlorophyll (*a* + *b*). S1: the undifferentiated stage, S2: the inflorescence primordium differentiation stage, S3: the inflorescence elongation stage, S4: the floret arrangement stage, S5: the anthesis stage. Values in the figure represent the mean ± SE (*n* = 3). Distinct letters indicate treatments that differ significantly (*p* < 0.05, Duncan’s test).

Chlorophyll, whose formation and accumulation are influenced by light quality, constitutes the material basis of plant photosynthesis, with chlorophyll *a* and chlorophyll *b* serving as the primary pigments. Chlorophyll content was measured in the leaves of *C. ensifolium* ‘Longyan Su’ across the six treatment groups (CK, R, 3R1B, 1R1B, 1R12B, B) that exhibited significant phenotypic differences (Figure 3).

Chlorophyll *a* and *b* were individually measured in the leaves of ‘Longyan Su’ under different treatments. It was revealed that the chlorophyll *a* content in the 1R12B and B treatments gradually decreased from the undifferentiated stage to the anthesis stage, while the R, 3R1B, and 1R1B treatments exhibited a trend of initial decrease followed by an increase. During the undifferentiated stage, the chlorophyll *a* content in both the 1R12B and B treatments was higher than that in CK. It indicated that blue light treatment was beneficial for promoting chlorophyll accumulation in *C. ensifolium* leaves during the vegetative growth phase, enhanced their photosynthetic capacity and thereby facilitated the floral transition. From the inflorescence primordia differentiation stage to the inflorescence elongation stage, chlorophyll *a* content was significantly increased under the 1R1B treatment, significantly decreased in the B treatment, and showed no significant change under the other treatments. At the floret arrangement stage, chlorophyll *a* content sharply increased in the R and 3R1B treatments, peaking at 0.96 mg·g^−1^ FW under the R treatment, which was significantly higher than that in CK. At this stage, the high chlorophyll *a* concentration enhanced photosynthesis during floral organ development, which promoted more nutrient accumulation and improved flower quality. In contrast, the pigment levels under B light treatment continued to decrease, remaining at low levels even during the period of flower opening (Figure 3A).

The chlorophyll *b* content of each treatment showed minimal variation throughout the flower bud differentiation period, but its response pattern to different treatments was similar to that of chlorophyll *a*. Compared with the control, blue light promoted the rapid synthesis of chlorophyll *b*, while red light favored the long term maintenance of chlorophyll content. In the B treatment, the peak chlorophyll *b* content occurred during the vegetative growth phase. The high concentration of chlorophyll promoted the accumulation of photosynthetic products and provided the material basis for the earlier flowering of *C. ensifolium*. In contrast, in the R and 3R1B treatments, the peak occurred during the floret arrangement stage, where sufficient nutrients ensured the improvement of flower quality (Figure 3B). In summary, the effects of different light quality treatments on chlorophyll *a*, chlorophyll *b*, and total chlorophyll content showed generally consistent trends.

### 2.4. Effects of Light Quality on Soluble Protein and Soluble Sugar in C. ensifolium Leaves

**Figure 4:**
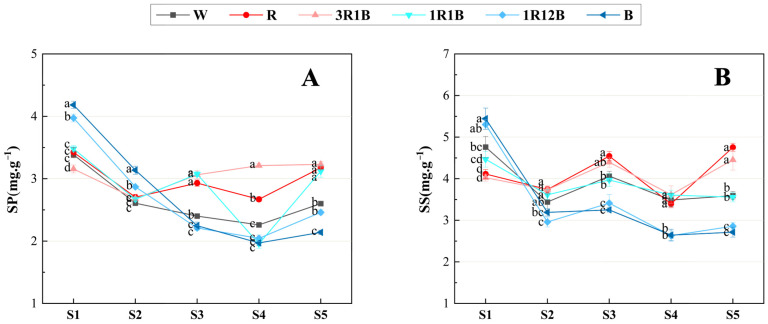
Effects of different treatments on the soluble protein and soluble sugar content in the leaves of *C. ensifolium* ‘Longyan Su’. (**A**) Soluble protein, (**B**) Soluble sugar. S1: the undifferentiated stage, S2: the inflorescence primordium differentiation stage, S3: the inflorescence elongation stage, S4: the floret arrangement stage, S5: the anthesis stage. Values in the figure represent the mean ± SE (*n* = 3). Distinct letters indicate treatments that differ significantly (*p* < 0.05, Duncan’s test).

Soluble proteins serve as both signaling molecules and structural components during flower bud differentiation in plants, and their accumulation contributes to the flowering process [20]. Soluble protein content was measured in the leaves of *C. ensifolium* ‘Longyan Su’ across the six treatment groups (CK, R, 3R1B, 1R1B, 1R12B, B) that exhibited significant phenotypic differences (Figure 4A).

During the whole process from the undifferentiated stage to flowering, the soluble protein content in the leaves of each treatment group generally exhibited an initial decrease followed by a slight recovery. From the undifferentiated stage to the inflorescence primordia differentiation stage, the soluble protein content in the leaves of the 1R12B and B treatments was significantly higher than that in other treatments, leading to an earlier onset of flower bud differentiation. At the inflorescence elongation stage, soluble protein content decreased in 1R12B and B, but increased in R, 3R1B, and 1R1B treatments, and at this stage, the R, 3R1B, and 1R1B treatments exhibited significantly higher protein levels than other treatments. From the inflorescence elongation stage to the floret arrangement stage, the soluble protein content in the R and 3R1B treatments remained significantly higher than that in CK, while the 1R1B treatment experienced a sharp decrease, reaching its lowest point (1.92 mg·g^−1^ FW) at the floret arrangement stage. At the anthesis stage, the soluble protein content in the 3R1B treatment was the highest among all treatments (3.23 mg·g^−1^ FW).

As direct products of photosynthesis, carbohydrates provide energy for plant flower bud differentiation and flowering processes [21]. Soluble sugar content was measured in the leaves of *C. ensifolium* ‘Longyan Su’ across the six treatment groups (CK, R, 3R1B, 1R1B, 1R12B, B) that exhibited significant phenotypic differences (Figure 4B).

Under different treatments, the soluble sugar in the leaves of *C. ensifolium* generally exhibited a “W” shaped variation trend throughout the whole differentiation process. During the undifferentiated stage, the soluble sugar content in the 1R12B and B treatments was relatively high, with the B treatment reaching the highest level (5.44 mg·g^−1^ FW), showing a significant increase compared with CK (4.76 mg·g^−1^ FW), indicating that blue light promoted an increase in soluble sugar in leaves during the undifferentiated stage. At the inflorescence primordia differentiation stage, the soluble sugar content in both the 1R12B and B treatments decreased sharply, with the 1R12B treatment showing the lowest content (2.96 mg·g^−1^ FW), significantly lower than that in CK (3.44 mg·g^−1^ FW). In contrast, the R, 3R1B, and 1R1B treatments showed slower nutrient consumption and maintained higher levels. During the mid-to-late stages of flower bud differentiation, two minor peaks emerged. The first occurred during the inflorescence elongation stage, where the R treatment reached its highest content (4.54 mg·g^−1^ FW), significantly higher than that in CK (4.06 mg·g^−1^ FW). While the 1R12B and B treatments showed significantly lower levels than that in CK. From the inflorescence primordia differentiation stage to the anthesis stage, the soluble sugar content in the R and 3R1B treatments consistently exceeded that of other treatments. The second “minor peak” occurred at the anthesis stage, at which time the R treatment (4.75 mg·g^−1^) reached its peak value throughout the entire flower bud differentiation period, significantly higher than that in CK (3.59 mg·g^−1^ FW). The 1R12B and B treatments exhibited continuous nutrient depletion, with nutrient contents significantly lower than those in CK at the anthesis stage.

### 2.5. Effects of Light Quality on the Endogenous Hormone Content in C. ensifolium Leaves

**Figure 5:**
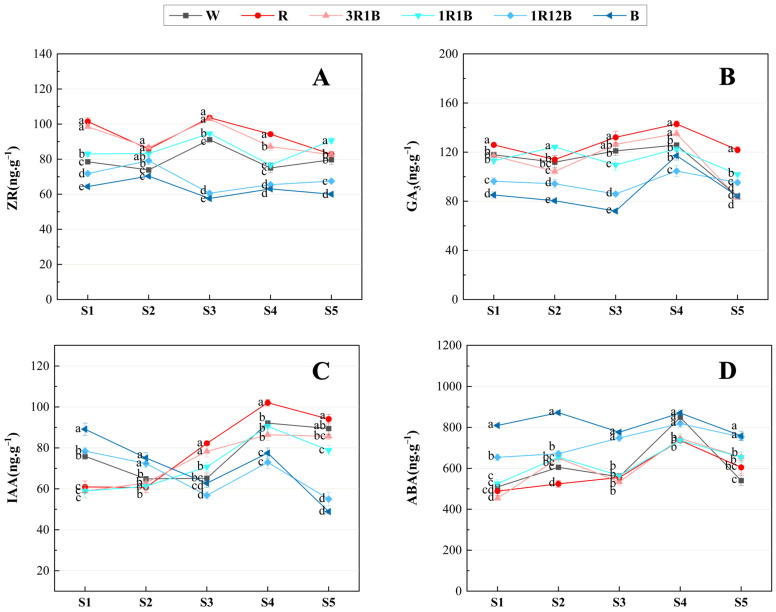
Effects of different treatments on the hormone content in the leaves of *C. ensifolium* ‘Longyan Su’. (**A**) zeatin riboside (ZR), (**B**) gibberellic acid (GA_3_), (**C**) indole-3-acetic acid (IAA), (**D**) abscisic acid (ABA). S1: the undifferentiated stage, S2: the inflorescence primordium differentiation stage, S3: the inflorescence elongation stage, S4: the floret arrangement stage, S5: the anthesis stage. Values in the figure represent the mean ± SE (*n* = 3). Distinct letters indicate treatments that differ significantly (*p* < 0.05, Duncan’s test).

The levels of endogenous hormones (IAA, ZR, GA_3_, and ABA) were measured in the leaves of *C. ensifolium* ‘Longyan Su’ across the six treatment groups (CK, R, 3R1B, 1R1B, 1R12B, B) that exhibited significant phenotypic differences. It was found that under treatments with a high red light ratio, the contents of ZR and GA_3_ were consistently and significantly higher than those under treatments with a high blue light ratio (Figure 5).

ZR is a type of cytokinin that promotes cell division and flower bud differentiation, as well as enhances plant resistance to stress [22]. The trends of ZR content variation under different treatments were inconsistent. The R, 3R1B, and 1R1B treatments showed trends similar to CK, exhibiting an initial increase followed by a decrease after the inflorescence primordia differentiation stage, with a peak occurring during the inflorescence elongation stage. At this stage, ZR content was significantly higher in the R and 3R1B treatments compared with CK. Subsequently, the ZR content in the R and 3R1B treatments continued to decrease until reaching the lowest point at the anthesis stage, while The contents of 1R1B and CK decreased during the floret arrangement stage and increased again during the anthesis stage. In contrast, 1R12B and B treatments exhibited an increasing-decreasing-increasing-decreasing trend, with the lowest ZR content occurring during the inflorescence elongation stage. The above results indicated that red and blue light treatments exerted distinct regulatory mechanisms on ZR metabolism of *C. ensifolium*. Red light significantly increased the ZR content in the leaves, while blue light inhibited the accumulation of ZR. Furthermore, higher concentrations of ZR had a positive effect on flower quality (Figure 5A).

GA_3_ is one of the most widely used gibberellins and is commonly applied to induce the floral transition in plants [23]. Determination of GA_3_ concentration in the leaves of *C. ensifolium* under different treatments revealed that the GA_3_ content generally followed a decreasing-increasing-decreasing trend during the process. The R and 3R1B treatments exhibited similar trends to CK, with continuous accumulation of content after the floral transition, peaking at the floret arrangement stage, where GA_3_ levels were significantly higher than that in CK. The 1R12B and B treatments began to decrease from the undifferentiated stage, reached the lowest value at the inflorescence elongation stage, and then gradually increased as flower development progressed. All treatments reached their peak during the floret arrangement stage, followed by a sharp drop at the anthesis stage. During the phase of flower bud differentiation, the GA_3_ level in plants continuously decreased, indicating that lower GA_3_ concentrations in leaves promoted the floral transition in *C. ensifolium*. From the inflorescence elongation stage to the floret arrangement stage, the concentration of GA_3_ gradually increased, indicating that higher GA_3_ levels were beneficial for the formation of floral organs, with red light promoting its accumulation during this stage and thereby improving flowering quality (Figure 5B).

IAA is the first identified hormone and one of the most important plant hormones [24]. For this study, the trend of IAA variation under different treatments was similar to that of GA_3_, both showing a decreasing-increasing-decreasing trend. During the flower bud differentiation stage, the IAA content remained relatively stable in the R, 3R1B, and 1R1B treatments, whereas it showed a decreasing trend in CK, 1R12B, and B treatments. After the completion of differentiation, the IAA level in CK, R, 3R1B, and 1R1B treatments gradually rebounded, reaching a peak at the floret arrangement stage. At this stage, the R treatment showed the highest IAA content (102.05 ng·g^−1^ FW), which was significantly higher than that in CK. This indicated that red light could promote IAA accumulation during the floret arrangement stage. In contrast, the 1R12B and B treatments showed a continuous decrease in content from the undifferentiated stage, reaching a minimum during the inflorescence elongation stage, with a brief rebound at the floret arrangement stage. At the anthesis stage, the IAA content in all treatments decreased to varying degrees, but the R treatment maintained a relatively high IAA level, while the IAA content in the 1R12B and B treatments dropped to the lowest point. The above results indicated that during the flower bud differentiation stage, different light quality treatments promoted IAA degradation, with degradation accelerating as the proportion of blue light increased. During flower development, red light promoted the accumulation of IAA, whereas blue light inhibited IAA synthesis, and the insufficient IAA content led to poor flower quality (Figure 5C).

ABA is a growth inhibitor that can suppress vegetative growth, regulate dormancy, and indirectly affect flower bud differentiation [25]. The determination of ABA content in each treatment revealed an overall “M”-shaped trend throughout the treatment process. Additionally, ABA content under the high blue light ratio treatments remained consistently higher than that in CK. Two minor peaks emerged during the process, with the first occurring during the inflorescence primordia differentiation stage. From the undifferentiated stage to the inflorescence primordia differentiation stage, ABA content continuously increased across all treatments, with the highest concentration observed in the B treatment (872 ng·g^−1^ FW), showing a significant difference from the CK. Following a brief decrease during the inflorescence elongation stage, ABA content in all treatments reached a second peak during the floret arrangement stage. At the anthesis stage, ABA content decreased across all treatments, with the CK exhibiting the lowest level (540.27 ng·g^−1^ FW). These results suggested that both red and blue light promoted the accumulation of ABA, with blue light exerting a stronger effect than red light (Figure 5D).

### 2.6. Effects of Light Quality on Endogenous Hormone Ratios in C. ensifolium Leaves

**Figure 6:**
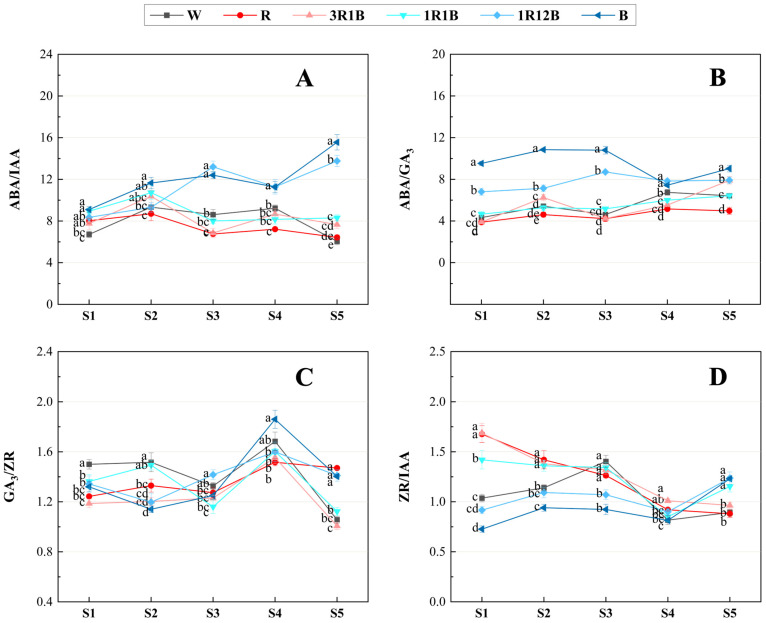
Effects of different treatments on endogenous hormone ratios in the leaves of *C. ensifolium* ‘Longyan Su’. (**A**) ABA/IAA, (**B**) ABA/GA_3_, (**C**) GA_3_/ZR, (**D**) ZR/IAA. S1: the undifferentiated stage, S2: the inflorescence primordium differentiation stage, S3: the inflorescence elongation stage, S4: the floret arrangement stage, S5: the anthesis stage. Values in the figure represent the mean ± SE (*n* = 3). Distinct letters indicate treatments that differ significantly (*p* < 0.05, Duncan’s test).

Hormonal regulation of plant growth and development is not solely dependent on specific hormones at particular developmental stages; rather, the dynamic balance among hormones often exerts a more significant influence on flower bud differentiation and floral organ development, although the balance of endogenous hormones varies across different plant species. Different treatments exerted varying effects on the ratios of endogenous hormones in the leaves of *C. ensifolium* ‘Longyan Su’ during the flower bud differentiation and development (Figure 6).

Comprehensive analysis of the dynamics endogenous hormone ratios in *C. ensifolium* ‘Longyan Su’ leaves under different treatments revealed that from the undifferentiated stage to the inflorescence primordia differentiation stage, the ABA/IAA ratio significantly increased in all treatments except the R and 1R12B treatments, exhibiting a “minor peak” during the inflorescence primordia differentiation stage. During the flower development stage, the ABA/IAA ratio in the R, 3R1B, and 1R1B treatments, along with the CK, showed a gradual decrease, whereas the ratio in the 1R12B and B treatments continued to increase, with the peak delayed until the inflorescence elongation stage. From the floret arrangement stage to the flowering stage, the ratios of R, 3R1B, and 1R1B treatments remained relatively stable; CK decreased markedly; whereas the 1R12B and B treatments increased significantly. At the anthesis stage, the ratio in CK was the lowest among all treatments, indicating that both red and blue light treatments promoted an increase in the ratio, with higher proportions of blue light having a more pronounced effect. During the flower bud differentiation stage, blue light promoted the floral transition by elevating the ABA/IAA ratio, whereas a high ABA/IAA ratio during the flower bud development stage was unfavorable for floral organs development (Figure 6A).

The trend in the ABA/GA_3_ ratio was generally consistent with that of the ABA/IAA ratio, with the red light treatment maintaining a lower ratio than the blue light treatment throughout the process, indicating that blue light promoted an increase in the ABA/GA_3_ ratio, and the higher the proportion of blue light, the greater the ABA/GA_3_ ratio, while the red light treatment showed the opposite trend (Figure 6B).

The trends of the GA_3_/ZR ratio in *C. ensifolium* leaves differed among the various light quality treatments. During the flower differentiation stage, the GA_3_/ZR ratio increased significantly in the 1R1B treatment and decreased significantly in the 1R12B and B treatments, whereas the other treatments showed no significant differences, indicating that a lower GA_3_/ZR ratio promoted flower bud differentiation and that blue light treatment facilitated the early floral transition of ‘Longyan Su’ by reducing the GA_3_/ZR ratio in leaves. From the inflorescence primordium differentiation stage to the inflorescence elongation stage, the ratio in the 1R1B treatment decreased synchronously with CK, while the ratio in the 1R12B treatment increased significantly, and the ratios of the other treatments remained statistically unchanged. As flower development progressed, the ratios in all treatments increased significantly, reaching a peak at the floret arrangement stage, and then sharply decreased at the anthesis stage (Figure 6C).

The ZR/IAA ratio exhibited different trends under different treatments. From the undifferentiated stage to the inflorescence primordia differentiation stage, the ZR/IAA ratio in the R, 3R1B, and 1R1B treatments gradually decreased, whereas the 1R12B, B, and CK treatments exhibited completely opposite trends. From the inflorescence primordium differentiation stage to to the floret arrangement stage, the GA_3_/ZR ratio in all treatments, except for CK, showed a continuous decline and reached the lowest level at the floret arrangement stage, with the CK exhibiting the highest ratio during the inflorescence elongation stage, indicating that all light quality treatments could lower the ZR/IAA ratio at this stage, and the effect became more pronounced as the proportion of blue light increases. From the floret arrangement stage to the anthesis stage, the ratios in all treatments continuously increased, with those in the 1R1B, 1R12B, and B treatments being significantly higher than that in CK. These results suggested that a lower ZR/IAA ratio within a certain range could promote flower bud differentiation, while higher levels of ZR/IAA had a favorable effect on flower bud development (Figure 6D).

## 3. Discussion

### 3.1. Influence of Light Quality on Flowering Time and Flower Quality of C. ensifolium

Light quality exerts a regulatory effect on flower bud differentiation and flowering in plants. Plants are able to perceive light and transduce light signals through different photoreceptors, enabling them to respond effectively to changes in the light environment. Among them, blue light primarily promotes flowering via photoreceptors such as CRY1 and CRY2, whereas red light mainly suppresses flowering through photoreceptors including PHYB, PHYD, and PHYE [26]. A large number of studies have shown that light quality is a key factor affecting the flowering process of plants. Currently, studies on light quality regulation of flower bud differentiation have primarily focused on model plants such as *Arabidopsis thaliana* [27].

Our study demonstrated that red and blue light treatments affected the flowering time and flower quality of the *C. ensifolium* ‘Longyan Su’. Compared with the CK, the blue light treatment advanced the flowering time but had no significant effects on flower scape length and flower quality; the red light treatment resulted in the best flower scape length, flower quality, and the total number of flowers in ‘Longyan Su’, though flowering occurred later. This demonstrates that blue light has a positive effect on advancing the flowering time, while red light has a role in improving flowering quality. Numerous studies have demonstrated that blue light, similar to far-red light, can effectively promote flowering [28]. For example, blue light promoted the early bud sprouting and flowering in *Petunia × hybrida* [29]; *Hippeastrum hybridum* flowered earlier under an R:B ratio of 1:9, while an R:B ratio of 9:1 promoted both vegetative and reproductive growth but delayed flowering [30]; *Chrysanthemum morifolium* [31] and *Arabidopsis thaliana* [32] exhibited earlier flowering under blue light, while red light inhibited flowering. These observations correspond with the findings reported in this study. The promotion of earlier flowering in ‘Longyan Su’ under blue light may be related to the cryptochrome mediated blue light signaling pathway. Blue light can inhibit the activity of the CONSTITUTIVELY PHOTOMORPHOGENIC 1 (COP1)/SUPPRESSOR OF PHYTOCHROME A (SPA) complex through cryptochromes, which increases the stability and accumulation of ELONGATED HYPOCOTYL5 (HY5). As an important transcription factor in light signal transduction, HY5 activates the expression of FLOWERING LOCUS T (*FT*) and SUPPRESSOR OF OVEREXPRESSION OF CONSTANS 1 (*SOC1*)*,* which ultimately promotes the floral transition. This signaling network has been well studied in *Arabidopsis* [33]. In contrast, phytochrome B is activated under red light and induces the inactivation of phytochrome interacting factors (PIFs) transcription factors such as phytochrome interacting factor 4 (PIF4) and phytochrome interacting factor 5 (PIF5) [34]. PIF4 is a positive regulator of *FT*, and reduced PIF4 activity is usually associated with delayed flowering [35]. In addition, PIF5 can influence GA and ABA levels through SOMNUS [36]. Therefore, the delayed flowering and improved flower quality of ‘Longyan Su’ under red light may be linked to changes in PIF4 and PIF5 activity and the participation of the GA signaling pathway.

### 3.2. Influence of Light Quality on Soluble Protein and Soluble Sugar Contents in Leaves of C. ensifolium

The growth, development, and flowering process of plants are closely related to the content of nutrients. It is found that light quality can regulate the metabolism of biomass such as carbohydrates and proteins in plants [37], change the content of carbohydrates through photosynthesis of plants [38], thereby affecting the flowering process and the quality of the flowers.

In this study, the contents of soluble protein and soluble sugar in all treatments showed a decreasing trend before and after the floral transition, which is consistent with previous findings [39]. A large number of nutrients were stored during the early stage of flower bud differentiation to provide energy for subsequent differentiation and development. At this stage, the leaves of ‘Longyan Su’ under blue light contained higher levels of soluble protein and soluble sugar, which provided a physiological basis for the floral transition. When the plants entered the flower development stage, the nutrient contents gradually decreased in all treatments. However, the nutrient levels under red light remained higher than those of the control and stayed elevated throughout this period. A possible explanation is that high levels of soluble protein and soluble sugar before inflorescence elongation favor the morphological development of flower buds. As floral development progresses, the formation of floral organs requires substantial nutrient consumption, which leads to a decrease in soluble protein and soluble sugar contents in the leaves. This conclusion is consistent with the results reported in *Cymbidium sinense* [40].

### 3.3. Influence of Light Quality on Hormone Contents in Leaves of C. ensifolium

The flower bud differentiation and flowering of plants are complex physiological processes [41], regulated by various internal and external factors. Plant hormones are key regulatory factors in the process of flower bud differentiation [42] and are commonly used as important substances for the artificial regulation of plant flowering [43]. Light quality influences hormone changes in plants through light signal transduction pathways. Photoreceptor related genes regulate the synthesis and metabolism of endogenous hormones and convey light signals to downstream gene networks, thereby influencing plant growth and development [44]. Among the phytochromes, PHYB is involved in the metabolic regulation of several plant hormones, and its role in the regulation of gibberellins is particularly important among the light induced flowering related hormones. In many model plants, blue light is often associated with the upregulation of gibberellin 2-oxidase (GA2ox) genes and a decrease in GA content. In contrast, red light commonly regulates the activity of transcription factors such as PIFs through PHYB and induces the upregulation of GA biosynthesis related genes, including gibberellin 3-oxidase (GA3ox) and gibberellin 20-oxidase 1 (GA20ox1), which increases GA levels and promotes the elongation of stems or flower scapes [45]. In addition, previous studies have suggested that red light facilitates the elevation of gibberellin (GA) and cytokinin (CTK) levels in plant leaves, whereas blue light favors the accumulation of ABA [46]. In this experiment, the content of ABA in *C. ensifolium* leaves increased under blue light treatment, and the contents of GA_3_ and ZR increased under red light treatment; this conclusion is consistent with the aforementioned research.

Blue light can regulate the balance among ABA, GA, and other hormones through the signaling pathway mediated by cryptochromes, thereby influencing flowering time [47]. The results of this study showed that before and after the floral transition, the endogenous ABA content continued to increase in all treatments, whereas the contents of IAA and GA_3_ decreased. Under blue light, the ratios of ABA/GA_3_ and ABA/IAA remained at relatively high levels, higher than those under the CK and red light treatments, indicating that an ABA dominated hormonal balance may be closely related to the promotion of floral transition in *C. ensifolium* ‘Longyan Su’. Previous studies have shown that high ABA levels can promote flower bud differentiation in plants such as *Prunus serrulata* [48] and *Camellia japonica* [49], and that ABA can regulate flowering time through multiple pathways, for example by inducing the early expression of *FT* and *SOC1*. In addition, blue light can regulate the biosynthesis and accumulation of GA, and other studies have reported that the advancement of flowering time under blue light is associated with reduced endogenous GA levels [50]. Moreover, ABA and GA often exhibit an antagonistic relationship during the reproductive growth stage in many plants [51]. Therefore, it is inferred that ABA might promote the floral transition by inhibiting the accumulation of GA_3_. In this study, ABA levels continuously increased and GA_3_ levels consistently decreased before and after the floral transition, leading to a gradual rise in the ABA/GA_3_ ratio. These findings indicate that under blue light, elevated ABA levels combined with reduced GA_3_ content are conducive to flower bud differentiation, which is consistent with the hormonal patterns reported in *Phalaenopsis* [39] and *Vanilla planifolia* [52].

During the flower development stage, the concentrations of GA_3_, IAA, and ABA in all treatments showed a gradual increasing trend. Compared with the control, red light significantly increased the levels of GA_3_, IAA, and ZR, while reducing ABA content and the GA_3_/ZR ratio. This change in hormone levels in *C. ensifolium* ‘Longyan Su’ was consistent with the results reported in *Lonicera japonica* [53] and *Allium sativum* [54]. Higher levels of ZR, GA_3_ and IAA promoted cell division and elongation, while lower levels of ABA helped reduce its inhibitory effect on floral organ growth, thereby facilitating the formation and development of floral organs. Previous studies have shown that red and blue light differ in their mechanisms of regulating flower scape cell activity. Blue light tends to suppress flower scape elongation by reducing cell number, whereas red light markedly promotes cell elongation, thereby increasing flower scape length [55]. Overall, red light modulates hormonal balance during the flower development stage by lowering ABA levels and enhancing the levels of ZR, GA_3_, and IAA, thereby laying the foundation for improved flower quality and flowering.

## 4. Materials and Methods

### 4.1. Experimental Material Schemes

The plant materials comprised healthy, pest-free, and uniformly grown three-year-old *C. ensifolium* ‘Longyan Su’ plants. All materials were cultured and maintained in our laboratory, and the plants were in the vegetative growth stage prior to treatment. The experiment was conducted in the Cangshan Campus of Fujian Agriculture and Forestry University, Fuzhou, China (Latitude: 26.061° N, Longitude: 119.312° E).

### 4.2. Experimental Treatments

**Figure 7:**
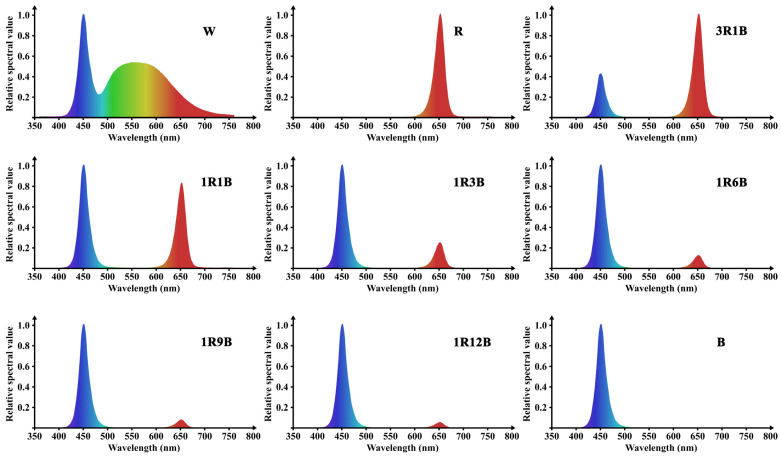
Relative spectra of different LED light quality treatments. Red represents red light (R), and blue represents blue light (B). The color gradient in the white light spectrum (W) visualizes the natural colors of visible light across wavelengths.

All plant materials were initially maintained under natural environmental conditions for 20 days and then transferred to a controlled-environment chamber equipped with LED lights. Three-year-old *C. ensifolium* plants were randomly assigned to 9 groups, each with 30 pots of 7 plants, in three replicates. They were placed in eight red-blue LED light zones and one white LED control zone (Table 1), with the corresponding spectral distributions presented in Figure 7. The light conditions were provided by flat-panel LED lamps designed by the research team of the College of Mechanical and Electrical Engineering, Fujian Agriculture and Forestry University. The light intensity and photoperiod were precisely adjusted and controlled using a control system manufactured by Xinchuang Moore Electronic Technology Co., Ltd. (Fuzhou, China). The photon flux density was measured using a Hipoint HR-350 spectrometer manufactured by HIPOINT Co., Ltd. (Taiwan, China). Plants were maintained at 80 ± 5 μmol·m^−2^·s^−1^ PPFD, 12 h·d^−1^ photoperiod, 75 ± 5% relative humidity, and 25 ± 2 °C in a controlled-environment chamber until the end of flowering period. To prevent interference between light sources that could affect the experimental results, all treatment groups were separated by black light-blocking fabric.

### 4.3. Recording of Flowering Time and Inflorescence Flowering Duration

Record the number of days required for flowering (from the beginning of treatment to the opening of the first flower on each plant), and the flowering duration of inflorescence (from the opening of the first flower to the fading of the last flower).

### 4.4. Measurement of Flowering Quality Indicators

Flowering traits were measured from treatment initiation until the end of flowering, with specific details and measurement methods outlined in Table 2.

### 4.5. Measurement of Plant Physiological Parameters

According to the previous classification of the stages of floral bud differentiation and development in *C. ensifolium* by our research group, five stages were selected: the undifferentiated stage (S1), the inflorescence primordium differentiation stage (S2), the inflorescence elongation stage (S3), the floret arrangement stage (S4), and the anthesis stage (S5). Leaf samples were collected from the same plant part of *C. ensifolium* in each treatment group at different developmental stages, rapidly frozen in liquid nitrogen and subsequently preserved at −80 °C. Three plants were randomly selected from each stage to serve as three biological replicates.

All physiological parameters in this study were measured from the leaves of *C. ensifolium* ‘Longyan Su’ plants. Chlorophyll content was determined following the ethanol extraction method [56]. Soluble protein and soluble sugar contents were quantified by the Coomassie Brilliant Blue G-250 assay [57] and the anthrone colorimetric method [58]. Endogenous hormones, including ABA, IAA, GA_3_, and ZR, were quantified by enzyme-linked immunosorbent assay (ELISA) using kits supplied by Shanghai Enzyme-linked Biotechnology Co., Ltd., Shanghai, China.

### 4.6. Statistical Analysis

Datas were analyzed by using one-way ANOVA in IBM SPSS Statistics 26.0 (IBM Corp., Armonk, NY, USA), and Duncan’s test was applied to assess the significance of differences among treatment groups (*p* < 0.05). Bar graphs were generated using GraphPad Prism 9.5.0 (GraphPad Software, San Diego, CA, USA), and line graphs using Origin 2021 (OriginLab Corp., Northampton, MA, USA). Values are expressed as mean ± standard error (SE), with error bars denoting the SE.

## 5. Conclusions

This research examined how light quality affects flowering and physiological parameters of *C. ensifolium* ‘Longyan Su’. Experimental findings revealed that blue light treatment significantly advanced the flowering time, promoting the accumulation of nutrients (soluble sugar and soluble protein) and endogenous hormones (ABA and IAA) in the leaves during the early stage of flower bud differentiation. In contrast, red light delayed flowering and improved flower quality, increasing the levels of nutrients (soluble sugar and soluble protein) and endogenous hormones (ZR, GA_3_, and IAA) during the flower development stage. Based on these results, red and blue light exhibited different regulatory patterns during the stages of flower bud differentiation and development of ‘Longyan Su’, providing a reference for the use of light quality to regulate flowering in *C. ensifolium*. For example, blue light treatment applied before flower bud differentiation can promote earlier flowering, whereas red light treatment applied at the same stage can delay flowering and improve flower quality.

## Figures and Tables

**Table 1:** LED light quality ratio treatments.

Treatments	Ratio (Red:Blue)	PPFD (μmol·m^−2^·s^−1^)	Photoperiod
W(CK)	white	80	7:00–19:00
R	red	80	7:00–19:00
3R1B	red/blue = 3:1	80	7:00–19:00
1R1B	red/blue = 1:1	80	7:00–19:00
1R3B	red/blue = 1:3	80	7:00–19:00
1R6B	red/blue = 1:6	80	7:00–19:00
1R9B	red/blue = 1:9	80	7:00–19:00
1R12B	red/blue = 1:12	80	7:00–19:00
B	blue	80	7:00–19:00

Notes: ‘W’ represents the control group (CK), ‘R’ represents red light (wavelength 660 nm), and ‘B’ represents blue light (wavelength 460 nm).

**Table 2:** Measurements of flowering traits.

Measurement Indicators	Specific Methods
Flower scape length/cm	The distance from the base of the pseudobulb to the tip of the flower
Flower scape diameter/mm	The diameter of the internode between the first and second flower from bottom to top
Flower transverse diameter/cm	The maximum horizontal width of the second flower from the bottom
Flower longitudinal diameter/cm	The maximum vertical length of the second flower from the bottom
Floret spacing/cm	The distance between the first flower and the second flower from bottom to top
Number of flowers/n	The total number of flowers on a single flower scape

## Data Availability

The original contributions presented in this study are included in the article. Further inquiries can be directed to the corresponding authors.

## References

[1] Ramya M., Park P.H., Chuang Y.C., Kwon O.K., An H.R., Park P.M., Baek Y.S., Kang B.C., Tsai W.C., Chen H.H. (2019). RNA sequencing analysis of *Cymbidium goeringii* identifies floral scent biosynthesis related genes. BMC Plant Biol..

[2] Di H., Zhao Y., Zhou A., Chen Z., Ma J., Liu D., Escalona V.H., Qian G., Yu X., Huang H. (2024). Integrated metabolome and transcriptome analysis revealed color formation in purple leaf mustard (*Brassica juncea*). Sci. Hortic..

[3] Galvão V.C., Fankhauser C. (2015). Sensing the light environment in plants: Photoreceptors and early signaling steps. Curr. Opin. Neurobiol..

[4] Ma Y., Xu A., Cheng Z. (2021). Effects of light emitting diode lights on plant growth, development and traits a meta-analysis. Hortic. Plant J..

[5] Lin K., Huang M., Hsu M. (2021). Morphological and physiological response in green and purple basil plants (*Ocimum basilicum*) under different proportions of red, green, and blue LED lightings. Sci. Hortic..

[6] Wang T., Sun Q., Zheng Y., Xu Y., Liu B., Li Q. (2024). Effects of Red and Blue Light on the Growth, Photosynthesis, and Subsequent Growth under Fluctuating Light of Cucumber Seedlings. Plants.

[7] Clack T., Mathews S., Sharrock R.A. (1994). The phytochrome apoprotein family in *Arabidopsis* is encoded by five genes: The sequences and expression of PHYD and PHYE. Plant Mol. Biol..

[8] Aalifar M., Aliniaeifard S., Arab M., Zare Mehrjerdi M., Dianati Daylami S., Serek M., Woltering E., Li T. (2020). Blue Light Improves Vase Life of Carnation Cut Flowers Through Its Effect on the Antioxidant Defense System. Front. Plant Sci..

[9] Ait-Ali T., Frances S., Weller J.L., Reid J.B., Kendrick R.E., Kamiya Y. (1999). Regulation of gibberellin 20-oxidase and gibberellin 3beta-hydroxylase transcript accumulation during De-etiolation of pea seedlings. Plant Physiol..

[10] Trouwborst G., Hogewoning S.W., van Kooten O., Harbinson J., van Ieperen W. (2016). Plasticity of photosynthesis after the ‘red light syndrome’ in cucumber. Environ. Exp. Bot..

[11] Wu W., Chen L., Liang R., Huang S., Li X., Huang B., Luo H., Zhang M., Wang X., Zhu H. (2024). The role of light in regulating plant growth, development and sugar metabolism: A review. Front. Plant Sci..

[12] Wojciechowska R., Hanus-Fajerska E., Kamińska I., Koźmińska A., Długosz-Grochowska O., Kapczyńska A. (2019). High ratio of red-to-blue LED light improves the quality of *Lachenalia* ‘Rupert’ inflorescence. Folia Hortic..

[13] Owen W.G., Meng Q., Lopez R.G. (2018). Promotion of Flowering from Far-red Radiation Depends on the Photosynthetic Daily Light Integral. HortScience.

[14] Heo J., Lee C., Hosakatte N., Paek K. (2003). Influence of light quality and photoperiod on flowering of *Cyclamen persicum* Mill. cv. ‘Dixie White’. Plant Growth Regul..

[15] Moradi S., Kafi M., Aliniaeifard S., Moosavi-Nezhad M., Pedersen C., Gruda N., Salami S.A. (2022). Monochromatic blue light enhances crocin and picrocrocin content by upregulating the expression of underlying biosynthetic pathway genes in saffron (*Crocus sativus* L.). Front. Hortic..

[16] Gharti Magar Y., Noguchi A., Furufuji S., Kato H., Amaki W. (2019). Effects of light quality during supplemental lighting on *Phalaenopsis* flowering. Acta Hortic..

[17] Li R., Li Z., Shang Z., Zhao L., Bai J., Wang Y. (2019). Effects of different light quality of LED on flowering of *Dendrobium officinale* Kimura et Migoin vitro. J. South. Agric..

[18] Guo Y., Zhong Y., Mo L., Zhang W., Chen Y., Wang Y., Chen H., Wang Z., Song X., Meng X. (2023). Different combinations of red and blue LED light affect the growth, physiology metabolism and photosynthesis of in vitro-cultured *Dendrobium nobile* ‘Zixia’. Hortic. Environ. Biotechnol..

[19] Deng J., Wang G., Yao C., Xing Q., Xie J., Huang Z., Pu S., Fan Y., Luo A. (2022). Effect of Light Quality on Morphological and Physiological Indexes of *Dendrobium denneanum*. J. Sichuan For. Sci. Technol..

[20] Zhang H., Wei Q., Li C., Jiang C., Zhang H. (2016). Comparative Proteomic Analysis Provides Insights into the Regulation of Flower Bud Differentiation in *Crocus sativus* L. J. Food Biochem..

[21] Zhong D., Ding M., Tang F., Mo R., Teng Y. (2013). Determination of Four Endogenous Phytohormones in Bamboo Shoots by Liquid Chromatography-Tandem Mass Spectrometry. Chin. J. Anal. Chem..

[22] Song G., Chen Q., Callow P., Mandujano M., Han X., Cuenca B., Bonito G., Medina-Mora C., Fulbright D.W., Guyer D.E. (2021). Efficient Micropropagation of Chestnut Hybrids (*Castanea* spp.) Using Modified Woody Plant Medium and Zeatin Riboside. Hortic. Plant J..

[23] Rademacher W. (2015). Plant Growth Regulators: Backgrounds and Uses in Plant Production. J. Plant Growth Regul..

[24] Lv B., Zhu J., Kong X., Ding Z. (2021). Light participates in the auxin-dependent regulation of plant growth. J. Integr. Plant Biol..

[25] Riboni M., Robustelli Test A., Galbiati M., Tonelli C., Conti L. (2016). ABA-dependent control of GIGANTEA signalling enables drought escape via up-regulation of FLOWERING LOCUS T in *Arabidopsis thaliana*. J. Exp. Bot..

[26] Putterill J., Laurie R., Macknight R. (2004). It’s time to flower: The genetic control of flowering time. BioEssays News Rev. Mol. Cell. Dev. Biol..

[27] Shibuya T., Kanayama Y. (2014). Flowering response to blue light and its molecular mechanisms in *Arabidopsis* and horticultural plants. Adv. Hort. Sci..

[28] Kong Y., Zheng Y. (2025). Complex Signaling Networks Underlying Blue-Light-Mediated Floral Transition in Plants. Plants.

[29] Fukuda N., Ajima C., Yukawa T., Olsen J.E. (2016). Antagonistic action of blue and red light on shoot elongation in *petunia* depends on gibberellin, but the effects on flowering are not generally linked to gibberellin. Environ. Exp. Bot..

[30] Wang S., Liu X., Liu X., Xue J., Ren X., Zhai Y., Zhang X. (2022). The red/blue light ratios from light-emitting diodes affect growth and flower quality of *Hippeastrum hybridum* ‘Red Lion’. Front. Plant Sci..

[31] Park Y.G., Jeong B.R. (2019). Night interruption light quality changes morphogenesis, flowering, and gene expression in *Dendranthema grandiflorum*. Hortic. Environ. Biotechnol..

[32] Tsukamoto A., Hirai T., Chin D.P., Mii M., Mizoguchi T., Mizuta D., Yoshida H., Olsen J.E., Ezura H., Fukuda N. (2016). The FT-like gene PehFT in *petunia* responds to photoperiod and light quality but is not the main gene promoting light quality-associated flowering. Plant Biotechnol..

[33] Hajdu A., Dobos O., Domijan M., Bálint B., Nagy I., Nagy F., Kozma-Bognár L. (2018). Elongated Hypocotyl 5 mediates blue light signalling to the *Arabidopsis* circadian clock. Plant J..

[34] Song Z., Heng Y., Bian Y., Xiao Y., Liu J., Zhao X., Jiang Y., Deng X.W., Xu D. (2021). BBX11 promotes red light-mediated photomorphogenic development by modulating phyB-PIF4 signaling. aBIOTECH.

[35] Kumar S.V., Lucyshyn D., Jaeger K.E., Alós E., Alvey E., Harberd N.P., Wigge P.A. (2012). Transcription factor PIF4 controls the thermosensory activation of flowering. Nature.

[36] Kim D.H., Yamaguchi S., Lim S., Oh E., Park J., Hanada A., Kamiya Y., Choi G. (2008). SOMNUS, a CCCH-type zinc finger protein in *Arabidopsis*, negatively regulates light-dependent seed germination downstream of PIL5. Plant Cell.

[37] Li H., Xu Z., Tang C. (2010). Effect of light-emitting diodes on growth and morphogenesis of upland cotton (*Gossypium hirsutum* L.) plantlets in vitro. Plant Cell Tissue Organ Cult..

[38] Zheng L., He H., Song W. (2019). Application of Light-emitting Diodes and the Effect of Light Quality on Horticultural Crops: A Review. HortScience.

[39] Dong F., Qi Y., Wang Y.N., Wang C.Z., Zhu J., Wang C.P., Ma L., Zhang J.H., Lv X.H. (2024). Screening of flower bud differentiation conditions and changes in metabolite content of *Phalaenopsis pulcherrima*. S. Afr. J. Bot..

[40] Li S. (2016). Mechanism of Flower Development and Early Flowering Technique of *Cymbidium sinense*. Master’s Thesis.

[41] Wang J., Luo T., Zhang H., Shao J., Peng J., Sun J. (2020). Variation of Endogenous Hormones during Flower and Leaf Buds Development in ‘Tianhong 2’ Apple. HortScience.

[42] Brenner W.G., Ramireddy E., Heyl A., Schmülling T. (2012). Gene regulation by cytokinin in *Arabidopsis*. Front. Plant Sci..

[43] Mornya P.M.P., Cheng F. (2018). Effect of Combined Chilling and GA_3_ Treatment on Bud Abortion in Forced ‘Luoyanghong’ Tree Peony (*Paeonia suffruticosa* Andr.). Hortic. Plant J..

[44] Kurepin L.V., Emery R.J., Pharis R.P., Reid D.M. (2007). Uncoupling light quality from light irradiance effects in *Helianthus annuus* shoots: Putative roles for plant hormones in leaf and internode growth. J. Exp. Bot..

[45] Zhao X., Yu X., Foo E., Symons G.M., Lopez J., Bendehakkalu K.T., Xiang J., Weller J.L., Liu X., Reid J.B. (2007). A study of gibberellin homeostasis and cryptochrome-mediated blue light inhibition of hypocotyl elongation. Plant Physiol..

[46] Aalifar M., Aliniaeifard S., Arab M., Mehrjerdi M.Z., Serek M. (2020). Blue light postpones senescence of carnation flowers through regulation of ethylene and abscisic acid pathway-related genes. Plant Physiol. Biochem..

[47] Kong Y., Zheng Y. (2025). Multiple Signals Can Be Integrated into Pathways of Blue-Light-Mediated Floral Transition: Possible Explanations on Diverse Flowering Responses to Blue Light Manipulation. Agronomy.

[48] Xu Y., Li J., Wang P., Wang W., Guo Y., Hao X., Du L., Zhou C. (2023). The Exploration of Flowering Mechanisms in Cherry Plants. Plants.

[49] Cai Y., Shi Z., Fu X., Zhao P., Tian M., Sun D., Wang J. (2024). Flower Bud Differentiation and Endogenous Hormone Changes of *Camellia* ‘High Fragrance’. Mol. Plant Breed..

[50] Gharti Magar Y., Ohyama K., Noguchi A., Amaki W., Furufuji S. (2018). Effects of light quality during supplemental lighting on the flowering in an everbearing strawberry. Acta Hortic..

[51] Lymperopoulos P., Msanne J., Rabara R. (2018). Phytochrome and Phytohormones: Working in Tandem for Plant Growth and Development. Front. Plant Sci..

[52] Zhuang H., Gu W., Wang H., Luo M., Zhao Q., Zhu Z., Xing Y., Song Y. (2018). Dynamic Changes of Endogenous hormones in *Vanilla* Leaf during Flower Bud Differentiation. Chin. J. Trop. Agric..

[53] Li J., Lian X., Wang L. (2019). Study on the Regulation Mechanism of Endogenous Hormones in Delayed Flowering of *Lonicera japonica*. Acta Hortic. Sin..

[54] Liu Z., Zeng L., Du X., Peng Y., Tao Y., LI Y., Qin J. (2024). Flower Bud Differentiation and Endogenous Hormone Changes of Rosa ‘Angela’. Bull. Bot. Res..

[55] Fukuda N., Nishimura S., Nogi M. (2002). Effects of localized light quality from light emitting diodes on geranium peduncle elongation. Acta Hortic..

[56] Taixiang X., Rongrong Y., Juan C., Lu C., Ye A. (2019). Photosynthetic Pigments Content and Chlorophyll Fluorescence Kinetics Parameters of a Leaf Mutant Cultivar of *Cymbidium ensifolium*. Subtrop. Plant Sci..

[57] Zahedi S.M., Hosseini M.S., Daneshvar Hakimi Meybodi N., Abadía J., Germ M., Gholami R., Abdelrahman M. (2022). Evaluation of drought tolerance in three commercial pomegranate cultivars using photosynthetic pigments, yield parameters and biochemical traits as biomarkers. Agric. Water Manag..

[58] Bahrololomi S.M.J., Raeini Sarjaz M., Pirdashti H. (2019). The effect of drought stress on the activity of antioxidant enzymes, malondialdehyde, soluble protein and leaf total nitrogen contents of soybean (*Glycine max* L.). Environ. Stress Crop Sci..

